# Selections

**Published:** 1860-09

**Authors:** 


### NEW FACTS IN REGARD TO GOLD.

At a recent meeting of the London Chemical Society, an interesting paper was read by Mr. Warrington, one of the members, in which was noticed a peculiar condition of some gold brought from Australia, that had caused considerable embarrassment. A large quantity sent from the Bank of England to the Mint was found to be singularly deficient in ductility, and it was so "rotten" that it could neither be rolled nor subjected to the usual processes of coining. There were about forty thousand ounces of the metal in this condition. A specimen of it was sent to Mr. Warrington, with instructions to endeavor to ascertain the cause of this rottenness, and, if possible, to devise a remedy. He found on analysis that the specimen contained .92 per cent. of silver, two per cent. of tin, and a like proportion of antimony. The presence of the latter metal was supposed to be the cause of the alteration in the usual mechanical character of the gold. and it was no easy matter to extract it mechanically, without loss of precious metal. After several experiments, Mr. W. found that oxide of copper, when melted with the gold, in the proportion of about ten per cent., removed the antimony, which then rose to the surface as a powder, and could be cleared off. When, however, this plan was attempted to be carried into operation on a large scale by the metallurgist at the Mint, it was not found to answer, and Mr. W. was again applied to. The gold had been melted in the usual black lead or plumbago crucibles, the carbon of which had combined with the oxygen, and thus prevented the proper action of the oxide of copper on the antimony. On the substitution, however, of clay crucibles for those of black lead, the process was found to be very efficacious, and the difficulty was removed.

The above is worthy the attention of the profession ; this "rottenness" or want of ductility in much of the gold used for manufacturing plate being well known and often complained of.

It is also an established fact that some of our gold, especially that of California, contains a proportion of antimony. If, therefore, antimony is the cause of this deficiency in gold, the above process for obviating the difficulty may be considered most valuable to all manufacturers of plate.

